# Inner Properties Estimation of Gala Apple Using Spectral Data and Two Statistical and Artificial Intelligence Based Methods

**DOI:** 10.3390/foods10122967

**Published:** 2021-12-02

**Authors:** Vali Rasooli Sharabiani, Sajad Sabzi, Razieh Pourdarbani, Mariusz Szymanek, Sławomir Michałek

**Affiliations:** 1 Department of Biosystems Engineering, Faculty of Agriculture and Natural Resources, University of Mohaghegh Ardabili, Ardabil 56199-11367, Iran; s.sabzi@uma.ac.ir (S.S.); r_pourdarbani@uma.ac.ir (R.P.); 2 Department of Machine Science, University of Life Sciences in Lublin, 20-950 Lublin, Poland; szymgm@wp.pl; 3 Department of Botany and Plant Physiology, University of Life Sciences in Lublin, 20-950 Lublin, Poland

**Keywords:** spectroscopy, artificial neural network, ripening, apple, non-destructive prediction, optimization algorithm

## Abstract

Fruits provide various vitamins to the human body. The chemical properties of fruits provide useful information to researchers, including determining the ripening time of fruits and the lack of nutrients in them. Conventional methods for determining the chemical properties of fruits are destructive and time-consuming methods that have no application for online operations. For that, various researchers have conducted various studies on non-destructive methods, which are currently in the research and development stage. Thus, the present paper focusses on a non-destructive method based on spectral data in the 200–1100-nm region for estimation of total soluble solids and BrimA in Gala apples. The work steps included: (1) collecting different samples of Gala apples at different stages of maturity; (2) extracting spectral data of samples and pre-preprocessing them; (3) measuring the chemical properties of TSS and BrimA; (4) selecting optimal (effective) wavelengths using artificial neural network-simulated annealing algorithm (ANN-SA); and (5) estimating chemical properties based on partial least squares regression (PLSR) and hybrid artificial neural network known as the imperialist competitive algorithm (ANN-ICA). It should be noted that, in order to investigate the validity of the methods, the estimation algorithm was repeated 500 times. In the end, the results displayed that, in the best training, the ANN-ICA predicted the TSS and BrimA with correlation coefficients of 0.963 and 0.965 and root mean squared error of 0.167% and 0.596%, respectively.

## 1. Introduction

The marketing can be managed by enhancing cost-effective and non-destructive quality control systems in food industries. The NIR spectroscopy has been successfully used to measure the physicochemical features of food and agricultural products non-destructively [1,2,3,4]. The internal properties of various fresh fruits have been successfully evaluated for several decades using NIR spectroscopy [5,6,7,8,9,10,11,12].

The results of a variety of spectroscopic and chemometric techniques have proven that NIR spectroscopy alone is quite effective for determining the physical and chemical properties of several fruits [13,14,15]. Other applied research in the field of spectroscopy that can be mentioned separately include: apple [16], sesame [17], pear [18], passion fruit [19], jujube [20], pomegranate [21], mango [2], grapes [22], and tangerine [19].

The feasibility of predicting the soluble solid content (SSC) of citrus was investigated by Tian et al. [23] using portable Vis/NIR spectroscopy (550–1100 nm). The original spectra was preprocessed to improve the data. The applications of Vis/NIR spectroscopy were reviewed by Li et al. [18] for quality evaluation of oilseeds. The ability of spectroscopy to identify the geographical origin of oilseeds and edible oils was studied. Maniwara et al. [19] estimated soluble solid contents (SSC), titratable acidity (TA), and the pulp content (PC) of purple berry fruit using NIR spectroscopy. They developed prediction models based on partial least squares (PLS) at the range of NIR spectrum. A non-destructive and rapid method was proposed by Xia et al. [17] to determine sesamin and sesamolin in sesame using NIR spectroscopy. Some sesame samples were collected from three different regions of China and the partial least squares (PLS) model. Huang et al. [24] used spatially resolved (SR) spectroscopy to evaluate the quality of tomatoes (at the range of 550–1650 nm). The results were obtained with two conventional single point spectroscopes (SP) at the range of 400 to 1000 nm and 900 to 1300 nm. The partial least squares (PLS) method was used to predict SSC and PH. A partial least squares model (PLS) was developed by Nascimentoa et al. [25] to measure the SSC and firmness of peach fruits with low and healthy frost, and to investigate the effect of maturity stage. FT-NIR spectra were obtained at three stages of maturity. They evaluated the performance of model through R^2^ and root mean squared error. The PLS method did not successfully classify fruits based on red and white skin color, maturity stages, and harvest season. A combination of a support vector machine with a feature extraction algorithm and X-ray computed tomography was presented by Looverbosch et al. [26] for the successful detection of internal malformations of pear. Wang et al. [27] evaluated kiwi fruit based on storage damage that is a physiological anomaly of colds. Water core is a symptom of this disease. Early signs appear only inside the fruit. Therefore, early detection is very helpful. There was a significant difference between damaged fruits and healthy fruits.

As obviously seen, various researches have been carried out on non-destructive methods to estimate the various physicochemical properties of fruits as well as their internal defects. Hence, this study is directed to present a non-destructive method based on hybrid ANN-ICA and PLSR using NIR spectroscopy to estimate properties of TSS and BrimA in Gala cultivar apples.

The innovations of the present study are (1) adjusting parameters of the artificial neural network optimally to guarantee high performance of the artificial neural network method to predict the properties of total soluble solids (TSS) and BrimA (TSS-K × TA); (2) selecting the optimal wavelengths using simulated annealing algorithm; and (3) performing the proposed algorithm for 500 times to assess the reliability of algorithm. The main difference between the current study with the other researches is that most of them either use linear statistical methods that use a simple ANN without any adjustment of parameters and so often gain errors in complex data. Moreover, in most researches, algorithms are executed only once; thus, they cannot achieve the same reliability.

## 2. Materials and Methods

**Figure 1:**
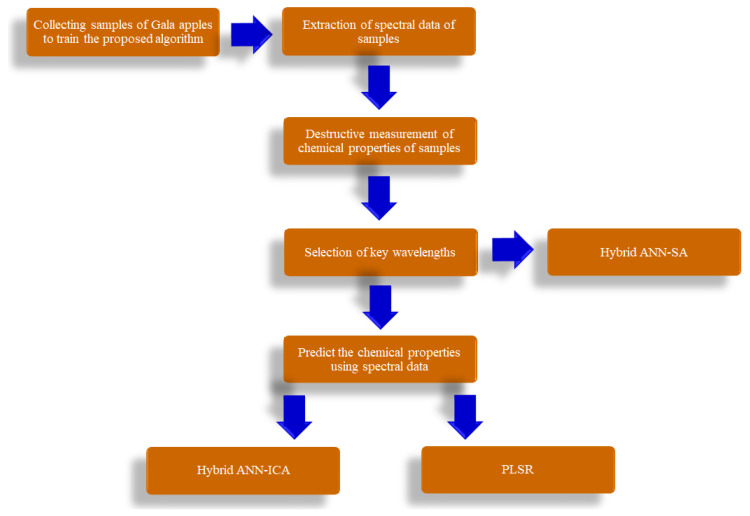
Schematic view of different steps of proposed algorithm in this research.

Figure 1 represents the different stages of different work stages of proposed method to predict TSS and BrimA of Gala apples. There are six main steps for training the proposed algorithm.

### 2.1. Collecting the Samples Used to Train the Proposed Algorithm

Gala apples were harvested in three different stages of their growth in different gardens in Karaj-Iran (located at latitude 35.83266 and longitude 50.99155). According to gardener’s experience, the harvest time of Gala apples were identified. Then, samples collected in 3 steps e.g., 14 days before harvesting time, at the time of harvest, and 7 days after that. At each step, 50 samples were collected and transferred to the laboratory for extracting the spectral data and measuring the chemical properties of TSS and BrimA.

### 2.2. Obtaining the Spectral Data

The hardware components for extracting spectral data includes a laptop (Intel Core i5, 500 M, 4 GB of RAM at 2.13 GHz, Windows 7) equipped with Spectra Wiz software, a spectrometer (EPP200NIR (StrllarNet, Tampa, FL, USA) in a 200–1100-nm region (VIS-NIR) with resolution of 2 nm, optical fiber and light source (tungsten halogen). Spectral data (5 scans per sample) were extracted from different intact apple samples. Finally, the average of these 5 scans was used for analysis. Spectral data often contain noise owing to various reasons, such as disturbing ambient light and unevenness of surface in apples [28]. Thus, in this study, three steps were used to generating exact data, namely conversion of reflectance spectra to absorption spectra by Equation (1), light scatter and baseline correction, and a smoothing operation using the wavelet filter [29].

Absorption spectra = log (1/reflectance spectra)(1)

### 2.3. Destructive Measurement of the Chemical Properties of TSS and BrimA

Sugars are the major soluble solids of fruit. As the fruit is ripe, the acid is converted to sugar and the accumulation of sugars in the fruit increases. Therefore, soluble solid contents typically determine the ripening and harvesting time of the crop. On the other hand, due to the effect of sugar and acid on the taste, the BrimA index is calculated based on the amount of soluble solids and acid. This index is also used to determine the time of ripeness in apples.

#### 2.3.1. TSS Content of Gala Apples

Since the amount of acid decreases and the amount of sugars increases with the ripening of fruits, therefore it is possible to identify the ripening stage of fruits, including Gala apples, using the values resulting from TSS determination that was applied by [30]. Normally, soluble solids determine the ripening and harvesting time of the crop. Soluble solids can be measured in a small sample of fruit extract by a refractometer. The refractometer shows the failure index. This indicator indicates the amount of reflected light rays after the light passes through the juice. Refractometers have a conversion scale and some others measure the amount of soluble solids in degrees of brix.

#### 2.3.2. Property of BrimA

This feature is used to grade the fruits based on taste and is used as an indicator to assess the ripening stage. This property is calculated by Equation (2) where TA is the titratable acidity. To measure titratable acid (TA), 5 mL of the extract obtained from the sample is taken and, for dilution, 45 mL of distilled water is added to it. The extract diluted with sodium hydroxide (NaOH) is titrated to 0.1 N, and the acid can be calculated using the Equation (3) in percentage.

The K indicates the sensitivity of the tongue to acid–sugar ratio which is usually considered to be 5 depending on the sugars and acids of individual fruit [31].

BrimA = TSS − K × (TA)(2)

(3) $$\mathrm{TA}=\frac{\mathrm{mL}\;\left(\mathrm{NaOH}\right)*N\left(\mathrm{NaOH}\right)*\mathrm{acid}\;\mathrm{meqfactor}*100}{\mathrm{mL}\;\mathrm{juice}}$$

### 2.4. Choosing the Key Wavelengths Using Hybrid ANN-SA Algorithm

The use of spectral data in the total range of 200–1100 nm has many limitations, which increases the computation complexity, due to high volume of data. On the other hand, the cost of development of portable devices and the computing time are the most important factors. Thus, determining effective and key spectral data should be helpful. In this study, the key wavelengths were selected by the ANN-SA algorithm. The simulated annealing algorithm (SA) simulates the annealing operation of metals. The annealing operation is needed to gain the least energetic and most stable state the material. The annealing operation will reach its goal whenever the temperature drop is slow enough. Therefore, first material is melted and then the temperature is decreased step by step until the material gets solid. In contrast, if material cools rapidly, the body will reach a near-optimal state that lacks the minimum energy [32]. In this study, a neural network was used to select effective wavelengths (Table 1). The input of this ANN involved various vectors of spectral data selected by a SA algorithm. The outputs of network are TSS and BrimA. Since mean squared error (MSE) of the neural network was calculated and saved for each vector, the ones with a lower MSE were introduced as key wavelengths.

### 2.5. Non-Destructive Estimation of TSS and BrimA

Two methods of ANN-ICA and PLSR were used to estimate properties of TSS and BrimA. It should be noted that, in order to assess the validation of the mentioned methods, the algorithm was executed for 500 times. In each iteration, 30% of the data were allocated to test data, 10% to validation data, and 60% to train data.

#### 2.5.1. Hybrid Artificial Neural Network-Imperialist Competitive Algorithm (ANN-ICA)

The multilayer perceptron artificial neural network has five adjustable parameters including the number of layers, the number of neurons, transfer function, back-propagation network training function, and back-propagation weight/bias learning function. The optimal adjustment of these layers guarantees high performance. In this paper, the imperialist competitive algorithm (ICA) has the task of optimally setting these parameters. ICA is an algorithm based on a mathematical model and simulation of human social and political evolution. The algorithm attempts to solve the problem by finding a general optimal point [33].

At first, the ICA algorithm includes a vector with a size equal to number of mentioned parameters. Each parameter is represented by a member. The mean squared error of vector was measured. If the mean squared error of vector was the lowest, it was assumed to be the most effective wavelength.

#### 2.5.2. Partial Least Squares Regression (PLSR)

As mentioned, the partial least squares regression is a non-parametric method that does not require data normalization [29]. Furthermore, despite the lost data, it has a high statistical power. This helps to explain several independent and dependent variables simultaneously.

### 2.6. Parameters Measuring the Performance of ANN-ICA and PLSR

To investigate the performance of ANN-ICA and PLSR, the evaluating criteria, namely the correlation coefficient (R), the coefficient of determination (R^2^), the mean squared error (MSE), the root mean squared error (RMSE), and the mean absolute error (MAE), were used [34].

## 3. Results

### 3.1. Optimally Tuned ANN Structure Based on ICA

The best structure of ANN to predict the chemical property of TSS and BrimA is given in Table 2.

### 3.2. Effective Wavelengths Selected by ANN-SA for Estimating TSS and BrimA

#### 3.2.1. Property of TSS

The wavelengths 953, 961, 977, and 983 nm were selected to estimate TSS of Gala apples by hybrid the ANN-SA method.

#### 3.2.2. Property of BrimA

The wavelengths, i.e., 958, 966, 972, 984 nm, were selected to estimate the BrimA of Gala cultivar by the hybrid ANN-SA method.

### 3.3. The Efficiency of ANN-ICA and PLSR Methods for Non-Destructive Prediction of TSS Based on Effective Wavelengths

#### 3.3.1. Propertiy of TSS

**Figure 2:**
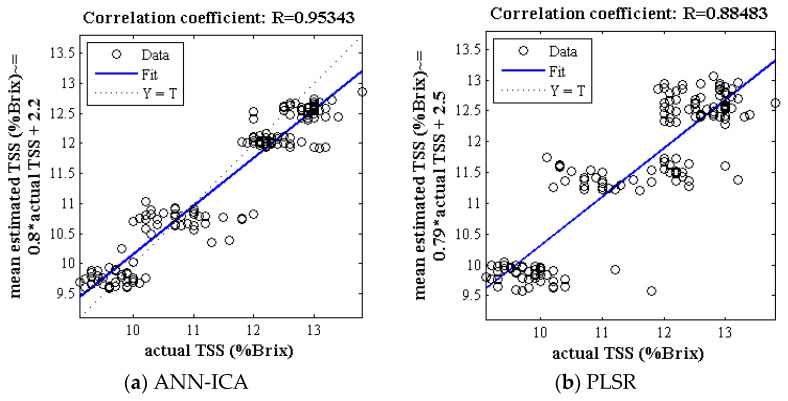
The correlation plot between mean predicted and true value of TSS of Gala cultivar (test set) for methods of ANN-ICA and PLSR. The multiply operation is denoted by the symbol *.

Figure 2 demonstrates the correlation plot between the mean predicted and true value of TSS of Gala cultivar (test set) by the ANN-ICA and PLSR classifiers based on spectral data in 500 iterations. The correlation coefficient for classifier ANN-ICA and PLSR is above 0.95 and 0.88, respectively, which is an acceptable value for estimating the TSS.

**Figure 3:**
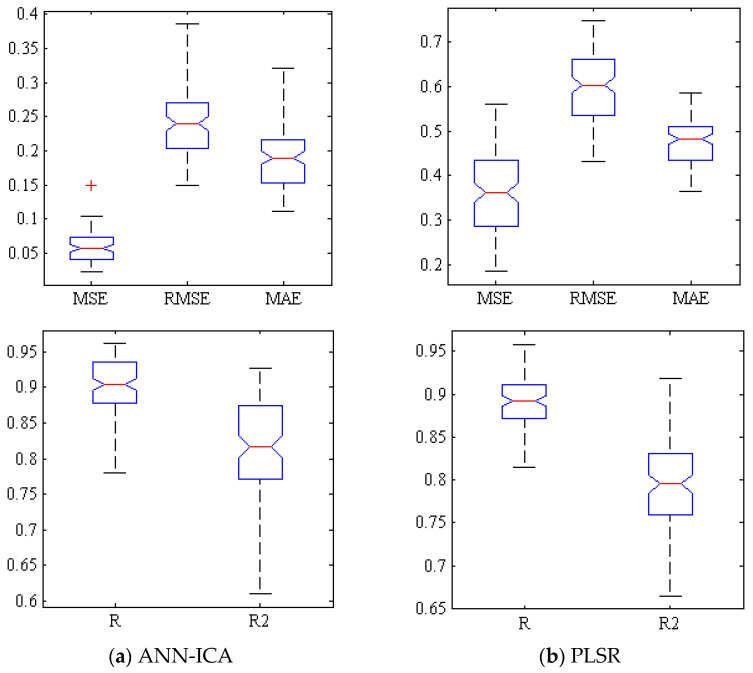
Box diagram of error criteria (1st row) and coefficients of correlation and determination (2nd row) related to (**a**) ANN-ICA and (**b**) PLSR in prediction of TSS.

Figure 3a shows a box plot of several criteria for evaluating the efficiency of ANN-ICA for predicting TSS within Gala cultivar based on spectral data of effective wavelengths at 500 iterations. Compact box diagrams mean that the results are close at different iterations. Among all iterations, only one of them has the mean squared error of greater than 0.1. Additionally, in more than half of the iterations, MSE values were less than 0.06 and the correlation coefficient was above 0.9. Figure 3b demonstrates the performance of PLSR method for predicting TSS. In most iterations, RMSE is more than 0.55 and less than 0.65. Furthermore, in the best state of training, this value is less than 0.45.

#### 3.3.2. Property of BrimA

**Figure 4:**
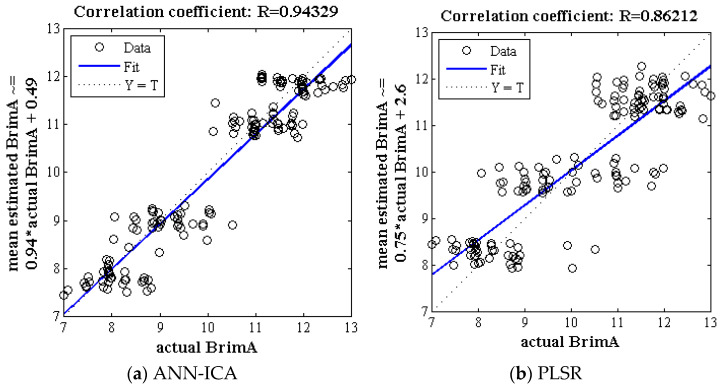
The correlation plot between mean predicted and true value of BrimA of Gala cultivar for classifiers of ANN-ICA and PLSR. The multiply operation is denoted by the symbol *.

Figure 4 shows the correlation plot corresponding to the mean predicted and true value BrimA by the ANN-ICA and PLSR algorithm in 500 iterations. According to Figure 4a, the value of the correlation coefficient obtained for classifier ANN-ICA is above 0.94, which is an acceptable value for non-destructive estimation. Figure 4b shows the correlation plot between mean predicted and true value of BrimA of Gala apples by PLSR method. The correlation coefficient obtained in this case is above 0.86.

**Figure 5:**
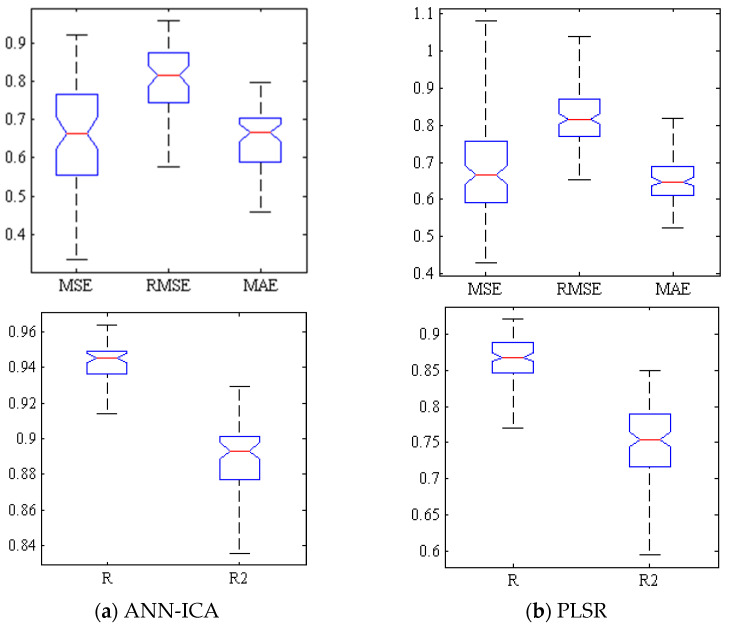
Box diagram of error criteria (1st row) and coefficients of correlation and determination (2nd row) related to ANN-ICA and PLSR in prediction of BrimA.

Figure 5 examines the performance of two classifiers, i.e., ANN-ICA and PLSR, using five criteria, including R, R^2^, MSE, RMSE, and MAE. Since in the error criteria, the distance between the first and middle quarters is less than the distance between middle and third quarters, so it can be concluded that, in most iterations, the results had a smaller error value.

### 3.4. Comparing the Efficiency of ANN-ICA and PLSR Methods for Predicting of TSS

Table 3 compares the efficiency of ANN-ICA and PLSR methods for predicting TSS of Gala cultivar in 500 iterations using mean and standard deviation. The ANN-ICA has lower values than the PLSR method in error criteria. Moreover, the coefficients of correlation and determination of ANN-ICA are higher than PLSR. Therefore, it can be concluded that the ANN-ICA method has a better efficiency than the PLSR method for predicting the non-destructive chemical property of TSS.

**Figure 6:**
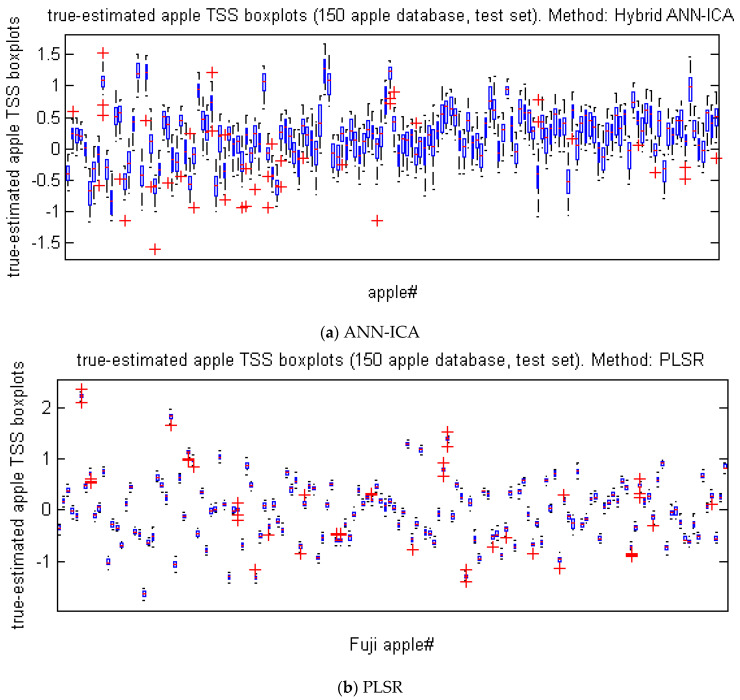
Box diagrams representing difference between the true and the predicted values of TSS in 500 replications. (**a**) ANN-ICA method, (**b**) PLSR method.

Figure 6 represents the box plot of the difference between the true (measured) and predicted values of TSS in 500 iterations to evaluate the efficiency of ANN-ICA and PLSR for predicting TSS of Gala apples. In fact, more compact box plot diagrams indicate that the predicted values are closer to the true values.

### 3.5. Comparing the Efficiency of ANN-ICA and PLSR for Prediction of BrimA

**Figure 7:**
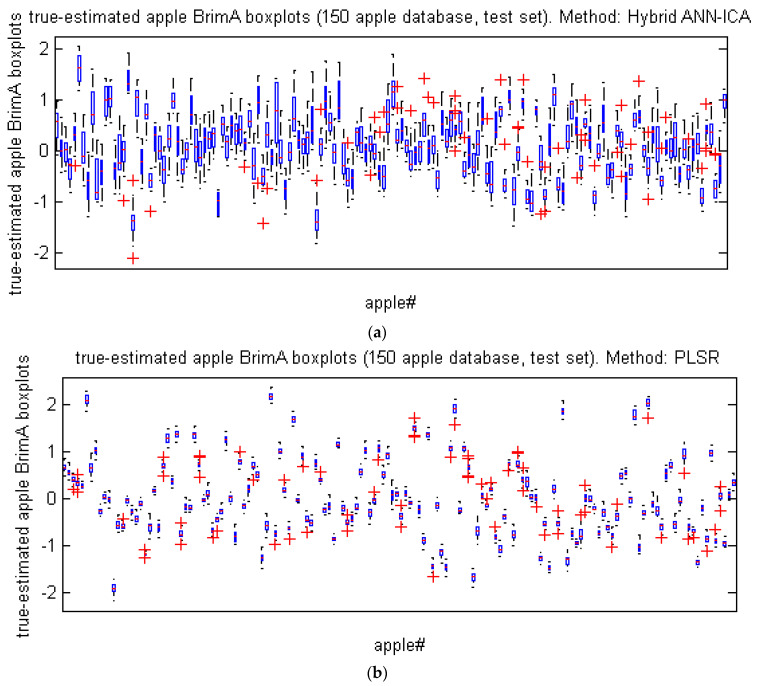
Box diagrams of the difference between the true and the predicted values of the BrimA of Gala apples in 500 iterations. (**a**) ANN-ICA method, (**b**) PLSR method.

Table 4 compares the efficiency of ANN-ICA and PLSR for predicting BrimA of Gala apple in 500 iterations using two criteria of mean and standard deviation. As can be seen, this method has a similar performance based on the error-related criteria. However, based on the coefficients of correlation and determination, the ANN-ICA method has a higher performance than the PLSR method. Figure 7 also examines the performance of these two methods based on a graphical analysis.

### 3.6. Comparing the Efficiency of Proposed Methods in This Study with Other Studies

The results represent that the proposed method in this article can predict the value of TSS content with correlation coefficient above 0.96. Tian et al. [23] and Maniwara et al. [19] proves this issue. Tian et al. [23] studied the prediction of TSS of citrus. Three apparent absorption peaks were found at 710, 810, and 915 nm in the original spectrum curve. Then, the key wavelengths were determined. Finally, prediction models were created based on entire wavelength and the key wavelengths. The results showed that the optimal prediction model had a correlation coefficient, a root mean squared error, and residual prediction deviation of 0.965, 0.584, and 512, respectively. The evaluation of purple berry fruit was conducted by Maniwara et al. [19] using NIR spectroscopy; the results indicated a significant relationship between the estimated and the actual values (0.84, 0.91, and 0.99 for SSC, TA, and PC, respectively).

## 4. Conclusions

Sugars are the major soluble solids in fruit. Herein, the present paper proposed the prediction of TSS and BrimA of Gala cultivar apples using ANN-ICA and PLSR based on spectral data related to key wavelengths. Low coefficient of determination in non-destructive estimation of soluble solids can be related to inappropriate spectral range, the effect of light scattering through changing the detector distance, change in sample size, surface roughness, noise caused by spectrometer temperature rise, software error, etc.

The most important results obtained from comparing two methods indicated that the ANN-ICA method with a correlation coefficient of 0.963 and an RMSE of 0.167 was able to predict the chemical property of TSS, while the correlation coefficient and RMSE of the PLSR method was 0.953 and 0.432, respectively. The same conclusion was made for property of BrimA.

In the end, it is suggested that the mentioned properties is predicted non-destructively at the range of 1000 to 2500 nm to be compared with the results of the present research. At higher wavelengths, the penetration of rays into the fruit is greater and provide more spectral information. So, new results may be obtained. Of course, it should be noted that developing a portable device based on wavelengths at the range of 200–1100 nm is less complex than wavelengths of 1000–2500 nm and will certainly be more practical.

## Figures and Tables

**Table 1:** The structure of ANN-SA for selecting the most effective spectrum.

Parameters	Specification
Number of neurons	1st layer: 13
	2nd layer: 23
Number of layers	2
Transfer function	1st layer: radbas
	2nd layer: logsig
Back propagation network training function	trains
Back propagation weight/bias learning function	learnh

**Table 2:** Structure of the hidden layers of ANN-ICA to predict TSS and BrimA.

Parameters	Specification	
	TSS	BrimA
Number of neurons	16, 20, 7	19, 23, 17
Number of layers	3	3
Transfer function	satlins, hardlim, tribas	netinv, logsig, logsig
Back propagation network training function	traincgb	traingd
Back propagation weight/bias learning function	learnsom	learnh

**Table 3:** Comparison of various criteria evaluating the efficiency of ANN-ICA and PLSR for predicting TSS in 500 replications using spectral data of key wavelengths.

Method	Criteria	MSE	RMSE	MAE	R	R^2^
Hybrid ANN-ICA	Mean and SD in 500 iterations	0.058 ± 0.022	0.237 ± 0.0466	0.186 ± 0.039	0.903 ± 0.037	0.818 ± 0.067
	In the best training	0.028	0.167	0.129	0.963	0.927
PLSR	Mean and SD in 500 iterations	0.366 ± 0.085	0.601 ± 0.071	0.473 ± 0.048	0.891 ± 0.027	0.794 ± 0.049
	In the best training	0.187	0.432	0.396	0.953	0.908

**Table 4:** Comparison of various criteria evaluating the efficiency of ANN-ICA and PLSR for predicting BrimA in 500 replications using spectral data of effective wavelengths.

Method	Criteria	MSE	RMSE	MAE	R	R^2^
Hybrid ANN-ICA	Mean and SD in 500 iterations	0.662 ± 0.139	0.809 ± 0.088	0.649 ± 0.081	0.943 ± 0.011	0.889 ± 0.019
	In the best training	0.355	0.596	0.487	0.965	0.931
PLSR	Mean and SD in 500 iterations	0.675 ± 0.128	0.818 ± 0.078	0.651 ± 0.064	0.866 ± 0.031	0.751 ± 0.053
	In the best training	0.475	0.693	0.549	0.922	0.851
